# Awareness of Nutrition and Supplements Among Pregnant and Preconception Women: A Real-World Study in Vietnam

**DOI:** 10.1089/whr.2023.0014

**Published:** 2023-10-25

**Authors:** Quang Thanh Le, Nguyen Khanh Trang Huynh, Thi Diem Tuyet Hoang

**Affiliations:** ^1^ Obstetrics Division, Tu Du Hospital, Ho Chi Minh City, Vietnam.; ^2^ Department of Obstetrics and Gynaecology, Pham Ngoc Thach University of Medicine, Ho Chi Minh City, Vietnam.; ^3^ Obstetrics and Genetics Department, Hung Vuong Hospital, Ho Chi Minh City, Vietnam.

**Keywords:** first 1000 days, health literacy, nutrition, preconception, pregnant, supplements

## Abstract

**Background:**

Few studies have addressed relationships between health literacy (HL) and nutritional awareness in preconception/pregnancy populations, especially within Asia. We explored the rationale for nutrition-related education and/or HL interventions to improve nutritional intake among preconception/pregnant women.

**Methods:**

A cross-sectional questionnaire-based real-world study was conducted among 100 preconception and 200 pregnant women in Vietnam in January/February 2022. The questionnaire included a validated screening tool for HL (Newest Vital Sign [NVS]), and questions on preconception/pregnancy-related nutritional knowledge and behavior, prenatal supplementation, sources of nutritional advice.

**Results:**

Most respondents (62%) had limited HL and only 5% had adequate HL. Respondents with limited HL (NVS 0–1) showed less awareness of benefits of healthy eating before/during pregnancy, such as reduction in risk of birth defects. Most (94%) considered prenatal supplements beneficial, yet 64% were not convinced of supplement safety. The limited HL group reported the lowest use of supplements, including multivitamins, iron, and folic acid/folate.

**Conclusion:**

The prevalence of limited HL and the low awareness of preconception/pregnancy-related nutrition suggest an urgent need to invest in nutrition-specific education and improving HL in maternal populations. This will help support adequate maternal nutrition and appropriate micronutrient supplementation before conception and throughout the “first 1000 days” of life.

## Introduction

The concept of the “first 1000 days,” from conception to a child's second birthday, as a critical early period that determines lifelong health was born from the Developmental Origins of Health and Disease framework. This concept of developmental programming describes how the early environment, especially nutrition, influences health and disease outcomes through the life-course.^1–6^ Because the child's brain, body, and immune system develop extremely rapidly during this critical early period,^1,3,7^ the availability of nutrients in the environment profoundly influences their survival and ability to grow, learn, and thrive.^3^

Adequate nutrition is crucial for maternal and child health, both before and during pregnancy. Malnutrition in pregnancy is associated with nutrient deficiency-related complications and outcomes for both mother and child, including maternal anemia, low birthweight, preterm birth, birth defects, and developmental delays.^2,8,9^ There is also growing evidence that various adverse outcomes, such as fetal malformations and spontaneous abortions, are more readily prevented by beginning interventions before conception.^10^ It is therefore widely advocated that interventions targeting maternal health and nutrition begin even before pregnancy, as part of preconception care.^10–12^ However, awareness of the importance of preconception health and nutrition appears low, resulting in missed opportunities to support proper growth and development by making nutritional, lifestyle, and other changes even before pregnancy is established.^13–15^

Maternal health literacy (HL) refers to the knowledge, skills, and confidence a woman has in understanding and managing her own health and that of her children.^16^ One key aspect of maternal HL involves awareness of good nutrition and its importance before and during pregnancy, and knowing how to ensure adequate and balanced nutritional intake. This includes understanding the role of specific micronutrients in conception and pregnancy, alongside other aspects of diet and lifestyle, such as avoiding alcohol and tobacco, and maintaining a healthy weight during pregnancy. Studies support the relevance of HL to self-care, health-directed behaviors, and health outcomes. For example, higher HL in pregnant women was found to be associated with positive prenatal self-care behaviors such as better nutrition, physical activity, and intake of dietary supplements.^17^ Another study found up to 50% reduction in the likelihood of severe stunting or severe underweight in children of women with higher versus lower HL.^18^

In Southeast Asia, inadequate or borderline dietary intake of micronutrients appears common among women of reproductive age,^19^ likely contributing to the higher prevalence of micronutrient-related adverse maternal and child health outcomes reported for this region.^20–22^ Strengthening maternal HL could help to increase the impact of existing interventions and programs for maternal and child health.

Although a relatively high prevalence of limited HL has been reported for Southeast Asian countries, including Vietnam,^23,24^ few or no published studies from this region have specifically examined the adequacy of HL in maternity populations in relation to nutritional awareness and behaviors during the conception/pregnancy journey. A real-world study was therefore conducted among preconception and pregnant women in Vietnam to investigate their levels of HL, awareness of preconception/pregnancy-related nutrition (including dietary intake and supplements), and associated behaviors during their conception/pregnancy journey.

## Methods

### Respondents and survey administration

This online cross-sectional questionnaire-based real-world study was conducted among preconception and pregnant women in Vietnam between January 28, 2022, and February 4, 2022. Respondents were recruited from an online consumer research panel managed by an established market research organization (Supplementary Data). Individuals may register to participate in such panels, which are selected to be representative of the included country or countries. Panel participants' data are secured and kept confidential by the organization managing the consumer panel. No personally identifying information is disclosed to external researchers using the panel, as only anonymized identifier codes are used for data collection. As an observational consumer research study, ethical approval was not required. Data collection and analysis were conducted by an independent research agency, IQVIA Solutions Asia Ltd, in accordance with locally applicable codes of conduct for consumer research.

Panel participants who expressed interest in this online survey were directed to provide informed consent and informed of the confidentiality of their data and opinions before accessing the survey. The survey took ∼20 minutes to complete. Respondents who completed the survey received a small reward in the form of “panel points” that can be accumulated and redeemed. The target sample size of 300 for this study (200 pregnant women, and 100 women who were planning to get pregnant within the next 12 months or were currently trying to conceive) was based on demographic and birth rate data for Vietnam. The estimated margin of error for the final sample of 300 was 6%.

For this study, the target population was specified as females aged 18–45 residing in 4 major urban centers in Vietnam (Ho Chi Minh City, Hanoi, Da Nang, Can Tho), who were planning to conceive or already pregnant. Survey recruitment involved a prescreening step and a quota system that was programmed to achieve the required sample size for each subgroup (exactly 200 pregnant women and 100 preconception women). Once these quotas were reached by collecting the required number of completed surveys, additional individuals attempting to complete the survey were screened out. The online survey form was programmed in a way that required respondents to answer all mandatory items (no missing items allowed).

### Questionnaire design

The Vietnamese-language questionnaire included questions about respondents' sociodemographic characteristics and conception/pregnancy journey, their nutritional knowledge and behavior changes related to conception/pregnancy, sources of nutritional advice, and perceptions regarding prenatal supplementation.

HL was assessed using the Newest Vital Sign (NVS) questionnaire, a practical tool to quickly screen for limited HL in health care and community settings.^25^ It was shown to be a reliable and valid HL measure, validated against standard HL assessments such as the Test of Functional Health Literacy in Adults.^25,26^ The NVS has been studied in several countries in general community samples^26–29^ and in pregnant women,^30^ and thus supports internationally comparable HL research. Literature searches conducted before the study did not identify any Vietnamese-language versions of the NVS that had been used or validated for HL screening in Vietnamese populations.

For this study, the English-language NVS questionnaire was therefore translated into Vietnamese by a native speaker, then checked for accuracy by native speakers using back-translation and comparison with the original questionnaire. The tool's brevity and its acceptability to research subjects made it highly suitable for use in the present study. Individuals are shown a food nutrition label and asked six questions to assess their ability to understand and appropriately interpret the text and numerical information on the label. Scores are based on the number of correct answers: high likelihood of limited HL (0–1), possibly limited HL (2–3), and adequate HL (4–6).

### Statistical analyses

Responses were summarized using descriptive statistics, including means and percentages. Selected items were further analyzed with respect to HL levels (limited HL, NVS score 0–1; possibly limited, score 2–3; adequate, score 4–6) or pregnancy status (pregnant/preconception). For between-group comparisons, *t*-tests were used to compare mean values, and *z*-tests were used to compare percentages. Significance testing of between-group differences was performed at the 5% level.

## Results

### Respondent characteristics

A total of 300 women completed the questionnaire; 200 in the pregnant group and 100 in the preconception group. Respondent characteristics are summarized in Table 1. The preconception respondents were older than the currently pregnant respondents.

**Table 1.** Demographics and Characteristics of Pregnant and Preconception Respondents

Characteristics	Pregnant (***N*** = 200), %	Preconception (***N*** = 100), %
Age, years		
18–24	8	10
25–29	51	27
30–34	34	38
35–39	6	14
40–45	3	11
Region of residence		
Ho Chi Minh City	49	46
Hanoi	45	35
Da Nang	6	9
Can Tho	1	10
Fertility treatments		
Yes	33	54
No	67	46
Pregnancy experience		
Yes	49	37
No	50	62
Prefer not to say	1	1
Planning status		
Planning pregnancy in 7–12 months	—	56
Planning pregnancy in the next 6 months	—	31
Currently trying to conceive a baby	—	13
Pregnancy stage		
First trimester	36	—
Second trimester	47	—
Third trimester	18	—

### HL levels

**FIG. 1.**
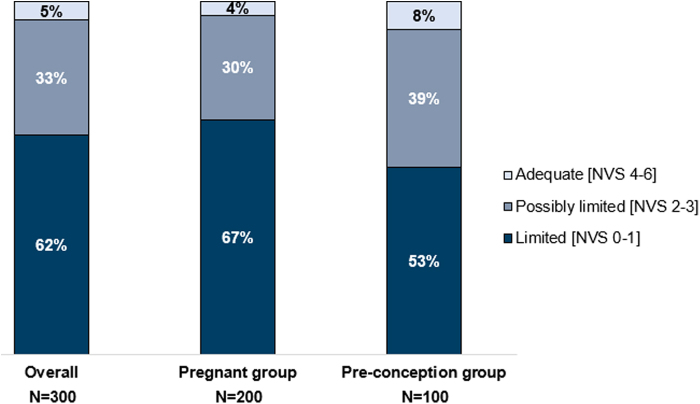
Health literacy level based on NVS scores. NVS, Newest Vital Sign.

The distribution of respondents' NVS score categories (HL level) is summarized in Figure 1. Out of 300 respondents, 62% had scores indicating a high likelihood of limited HL (0–1 correct answers), 33% had scores indicating possibly limited HL, and only 5% (16 respondents) had adequate HL. A higher proportion of pregnant women (67%) had scores indicating limited HL compared with preconception women (53%) (Fig. 1). Most respondents had at least a college or undergraduate education, medium or high household income, and were currently working. In this sample, HL did not show notable relationships with income level or employment status although the older respondents had higher HL levels (Supplementary Table S1).

### Knowledge and perceptions of nutrition in preconception/pregnancy

More than 95% of respondents considered healthy eating habits and improving their knowledge about healthy eating habits to be very or extremely important and around half of respondents (57% overall) rated their knowledge of healthy eating habits as excellent or very good (Supplementary Fig. S1). Interestingly, a high percentage (65%) of the respondents with limited HL (NVS 0–1) rated their knowledge of healthy eating habits as excellent/very good, which was higher than those with better HL scores (Supplementary Fig. S1B).

**FIG. 2.**
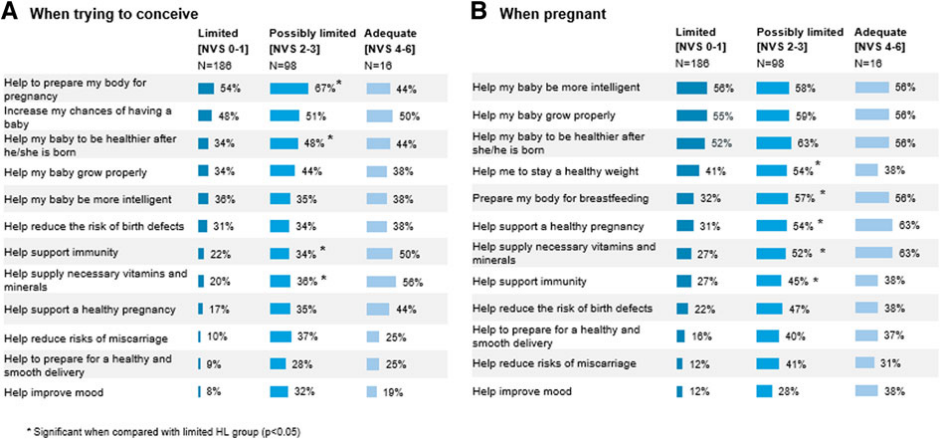
Knowledge of benefits of healthy eating when trying to conceive and when pregnant, by health literacy level. **(A)** When trying to conceive. **(B)** When pregnant.

When asked to identify specific benefits of healthy eating before and during pregnancy, respondents recognized only a few of the key benefits, and tended to focus more on healthy eating during pregnancy than before conception (Fig. 2). Notably, respondents with limited HL were less likely than those with higher HL to identify the various important benefits of healthy eating when trying to conceive and when pregnant. Overall, there was limited awareness that healthy eating before conception as well as during pregnancy can help to reduce the risk of miscarriage, improve mood, or help to prepare for a smooth delivery (Fig. 2). More than half of respondents (59%) reported some form of dietary restriction, including avoidance of dairy- or gluten-containing foods, or other restrictions (data not shown).

### Knowledge, perceptions, and usage of prenatal supplements

**FIG. 3.**
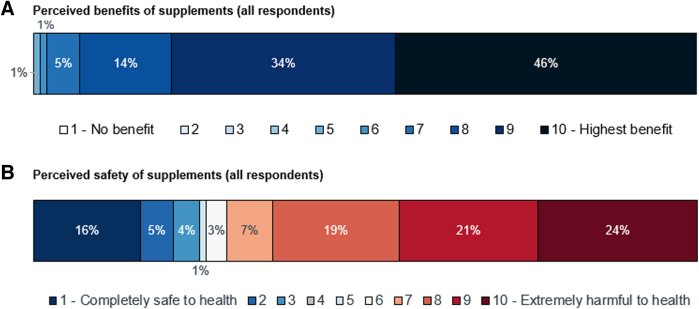
Perceptions of prenatal supplements. **(A)** Perceived benefits of supplements based on the response to the question: “On a scale of 1 to 10, how would you rate the overall benefit of nutritional supplements taken during preconception and pregnancy? (1—no benefit, 10—highest benefit).” **(B)** Perceived safety of supplements based on the response to the question: “On a scale of 1 to 10, how would you rate the overall safety of nutritional supplements taken during preconception and pregnancy? (1—completely safe to health, 10—extremely harmful to health).”

Most respondents recognized the benefits of taking nutritional supplements when trying to conceive or when pregnant, with 94% rating the benefits of supplements highly (≥8 of 10; 0—no benefit; 10—highest benefit) (Fig. 3A). On the contrary, 64% were not convinced of the safety of supplements, and perceived them as harmful (≥8 of 10; 0—completely safe; 10—extremely harmful) (Fig. 3B). A slightly higher proportion of respondents with limited HL (66%) rated supplements as harmful (≥8 of 10), compared with those who had possibly limited (59%) or adequate (63%) HL (data not shown).

**FIG. 4.**
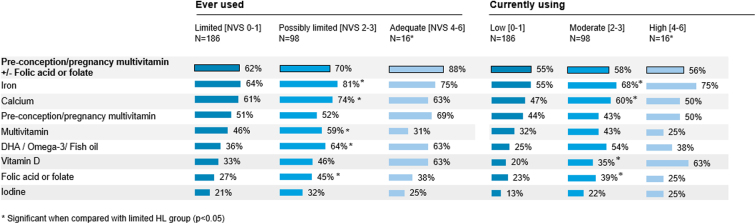
Current and past nutritional supplement usage by health literacy level. DHA, docosahexaenoic acid.

Less than half of respondents reported current use of preconception/pregnancy multivitamin supplements. Besides preconception/pregnancy multivitamins, iron and calcium were the most common supplements that respondents had ever used (Fig. 4). Less than half of respondents reported current use of folic acid/folate supplements.

Both current and past use of supplements tended to be higher among respondents with higher HL (Fig. 4). Only 55% of respondents in the limited HL group were currently supplementing iron, compared with 68% of those with possibly limited HL (*p* < 0.05). Similarly, only 23% of respondents with limited HL were currently using folic acid/folate supplements, compared with 39% of those with possibly limited HL (*p* < 0.05).

Compared with respondents in their first trimester of pregnancy, a higher proportion of respondents in their second and third trimester reported current use of supplements (Supplementary Fig. S2). Only 42% of respondents in their first trimester were taking preconception/pregnancy multivitamins with or without folic acid/folate, compared with 75% of those in their third trimester. Similarly, only 24% of respondents in their first trimester reported taking folic acid/folate supplements, compared with 47% of those in their third trimester, and the proportion taking preconception/pregnancy multivitamins was 30% in the first-trimester group and 64% in the third-trimester group.

### Sources of nutrition advice/information

For respondents, the most trusted sources of information about nutrition were health care professionals (HCPs) (77%), family or friends (47%), and websites (36%) (Supplementary Fig. S3). HCPs appeared to be the most trusted source of nutritional advice for women, regardless of HL level or pregnancy status. Respondents also noted that their HCPs provided advice on most areas, including nutrition, lifestyle changes, and prenatal supplements.

## Discussion

In this cross-sectional real-world study in Vietnam, a comprehensive questionnaire was used to characterize the awareness of preconception/pregnancy-related nutrition in women who were or planning to conceive or pregnant, and to explore how this awareness related to their HL level. Despite recognizing the general importance of nutrition for pregnancy, many respondents, especially those with limited HL, appeared to have an incomplete understanding of specific aspects of preconception/pregnancy-related nutrition, particularly its contributions to both maternal and child health outcomes. Moreover, with nearly two in three respondents having limited HL, there is a clear need to consider interventions to improve HL in maternity populations, in addition to knowledge-focused nutrition education.

### Limited HL and awareness of preconception/pregnancy-related nutrition

Although the women in this sample had relatively high levels of education and socioeconomic status, more than 60% of them had limited HL (NVS 0–1). Respondents with limited HL tended to overlook certain important benefits of healthy eating in pregnancy, such as supporting a healthy pregnancy and reducing the risk of birth defects, and helping their baby to be healthier after birth (Fig. 2). Despite these gaps in awareness, respondents with limited HL expressed as much or greater confidence in their knowledge of healthy eating than those with higher HL (Supplementary Fig. S1B), suggesting specific needs for engagement and education.

There are few studies of HL specific to the preconception/pregnancy populations in the region available for comparison, although it has been reported that HL levels in the general population in Southeast Asian countries are low.^23,24^ Our findings are reminiscent of those from a study of mothers with children <5 years in the urban and rural areas of Laos, where >80% of mothers had inadequate HL. In this study, maternal HL was associated with better health practices and health status.^31^

### Awareness and appropriate use of prenatal supplements

Prenatal micronutrient supplementation is recognized as one of several interventions that can improve maternal nutritional status and pregnancy outcomes.^32–34^ This is especially relevant in populations where underlying micronutrient deficiencies or insufficiencies are more common, as described for Asian women of reproductive age.^19^ A high proportion (59%) of respondents reported having dietary restrictions, which could make it challenging for them to consume a balanced diet that fully meets their nutritional requirements during pregnancy or while trying to conceive. There appeared to be limited awareness of the requirement for a range of micronutrients during the conception/pregnancy journey.

Although it is recommended to ensure adequate levels of 15 or more key micronutrients,^33–35^ respondents appeared to be focusing on a few micronutrients, such as iron and calcium. Slightly more than half of respondents were currently supplementing with a preconception/pregnancy multivitamin with or without folic acid/folate, and use of supplements was lowest among those with limited HL (Fig. 4).

The observed patterns of supplement use across stages of conception/pregnancy suggest that women might not be supplementing a number of micronutrients early enough in pregnancy. For example, folate is a key micronutrient during the first trimester, being required for proper neural tube development and to protect against congenital heart defects.^36^ Folate supplementation is therefore recommended for all pregnant women from before conception until at least the first trimester.^37^

However, less than half of respondents in their first trimester were currently taking a preconception/pregnancy multivitamin with or without folic acid/folate, compared with three-quarters of respondents in their last trimester (Fig. 1A). This is perhaps surprising, since more than 90% of respondents reported that they considered prenatal supplementation highly beneficial (Fig. 3A). A variety of factors might be contributing to the lower-than-expected rates of supplementation with folate/folic acid, whether as a component of a pregnancy multivitamin or a single supplement.

In Vietnam, it has been reported that relatively few women have their first antenatal care visit by the first trimester,^38^ and thus, since folate/folic acid containing supplements are usually prescribed by the attending HCP at the first antenatal visit,^39^ this could be one of a number of factors contributing to lower rates of supplementation in early pregnancy. It is also critical to ensure that the local practice setting is considered when following international or national-level recommendations, and that guidelines are reviewed regularly and kept up to date. Given the local context, recommendations for women to begin folic acid supplementation (400 μg/day) from the time they plan to conceive,^40^ rather than from the first antenatal visit, might help ensure consistent and timely folic acid supplementation.

Over the past decade, folic acid supplementation in pregnancy has not been strongly emphasized nationally in health promotion initiatives, such as the Vietnam National Nutrition Strategy for 2011 to 2020.^41^ This may contribute to low awareness of the need for folic acid/folate during pregnancy, and low prevalence of folic acid supplementation,^42^ consistent with what our data suggest.

Despite the availability of international and local recommendations on the micronutrient content and use of prenatal supplements to ensure nutritional adequacy and safety,^33–35^ we identified considerable ambivalence and strong negative perceptions among respondents concerning supplement safety. Although aware that prenatal supplements may be beneficial, respondents may not take them consistently or at the appropriate stages of their conception/pregnancy journey because of uncertainty about whether it is “safe” to do so. Concerns among the general public about food/supplement safety are understandable in jurisdictions with inadequate or poorly enforced regulations. However, this presents a problem if it leads to the misconception that all kinds of supplements, including important micronutrients, are unsafe, and that their consumption should be limited in terms of duration or amount. It is crucial to proactively address misconceptions that might discourage the use of prenatal supplements where needed.

These findings highlight the need for greater attention to planned preconception and early pregnancy care and education, including the importance of beginning supplementation early to establish adequate nutritional status well before pregnancy. Women may also benefit from having alternative sources of preconception education and information support available, especially if there are barriers to timely antenatal consultations when health advice is usually given.

### Strengthening parental HL for better health outcomes

Throughout the conception and pregnancy journey, parents frequently need to understand, weigh, and act on health-related information in various situations that influence health and pregnancy outcomes. In our study, the observed gaps in knowledge of appropriate nutrition and supplement use, coupled with the high frequency of limited HL, suggest that many women are not well prepared to manage nutrition optimally despite its importance for their health and that of the child. Considering the relatively high educational attainment of the sample, the low levels of HL may seem surprising. However, previous studies suggest that high educational attainment should not automatically be equated with high HL.^43^

The findings underscore the critical importance of general and nutrition-related HL in maternity populations. For example, improving HL could help to counter problematic practices and beliefs about pregnancy and childbirth that can lead to nutritional and health issues, such as the practice of limiting food intake to encourage a smaller baby and easier labor or, conversely, excessive weight gain from the practice of “eating for two” (reviewed in Ahmad et al.^44^ and Guggino et al.^45^).

As the most trusted sources of health-related information on conception, pregnancy, and childbirth (Fig. 3), HCPs are well placed to increase awareness and provide evidence-based nutritional advice. However, HCP recommendations do not always translate to improved HL and behavioral change, as noted in a study in Laos.^31^ In our study, almost all respondents reported receiving advice on diet/nutrition and prenatal supplements from their HCPs, yet their responses often indicated gaps in understanding or behaviors inconsistent with health recommendations. For HCPs, it may be helpful to assess the individual's HL directly during consultation with a brief screening tool, such as the NVS used in this study.

To engage women at all stages of preconception and pregnancy, HL-appropriate interventions involving a range of tailored communication strategies would be beneficial, but need not be delivered solely by HCPs. To further ease the burden on care providers, there can be greater utilization of other non-HCP resources, such as maternal health information resources and community programs. Through partnerships and resource-sharing, diverse stakeholders, including government bodies and private organizations, could contribute to such resources and help establish a supportive ecosystem for maternity and family care.

More broadly, efforts to improve HL in the general population could yield benefits for individuals as well as families and communities. There is also great potential for using mass media, such as television, to complement and reinforce health messages delivered by traditional means.^46^ A clear priority is to ensure that trustworthy and accurate information is available on such channels and platforms, and that it is accessible to individuals with different HL levels.

### Limitations

While this cross-sectional study yielded insights on the current level of HL among preconception/pregnant women in a real-world setting, and provided an in-depth exploration of respondents' knowledge of nutritional requirements and supplement usage during preconception and pregnancy, there are some limitations. First, the real-world setting necessitated the use of a brief HL screening tool (NVS). Since no studies have previously reported the use or validation of Vietnamese versions of the NVS in Vietnamese populations, the version used in the study was produced by professional translation of the English questionnaire. Additional testing/validation in Vietnamese populations and comparisons with other populations may be required. As a brief screening tool, the NVS is not capable of examining all possible dimensions of HL, which is a highly complex concept.^47^

Nevertheless, the finding of limited HL in this study clearly indicates important unmet needs, and additional research might be needed to understand whether particular aspects of HL should be addressed. These needs might include the ability to find, understand, judge, or apply health information, as well as the motivation to do so.^47^ We suggest that the form of quick screening performed here could help HCPs and other care partners to recognize signs of limited HL in typical care settings, for example, in conception planning or antenatal consultations. This may enable HCPs to communicate information in a way that the individual can understand.

Second, due to the lack of data on HL in maternal populations in Vietnam before our study, it was not possible to design the recruitment strategy to ensure a balance of responses at all HL levels. In our sample, only 16 of the 300 respondents (5%) had scores indicating adequate HL. Thus, we could not reliably examine the relationships between nutritional knowledge and behaviors across all levels of HL. Our findings may best describe the status of pregnancy-related nutritional knowledge and perceptions in Vietnamese women with limited or possibly limited HL. Although our NVS data are not directly comparable with available studies in Vietnam that used other HL instruments,^24^ there are published studies utilizing the NVS in other Asian countries such as Malaysia, China, and Taiwan.^28,29,48^

As specified by the research protocol, only Vietnamese participants in four large urban centers (Ho Chi Minh City, Hanoi, Da Nang, Can Tho) were included. However, the majority of Vietnamese citizens in these cities have internet access, and the participants fall within the typical age range for active internet users in the country. As such, no bias was expected from digital/online versus other modes of survey administration. However, we note that most of the respondents were working adults from major urban areas in Vietnam, well-educated, and with medium- to high-income levels, and thus, the results may not reflect the situation throughout the country. However, the results may allow for comparisons with groups of people with a similar socioeconomic and demographic profile in other Asian countries.

## Conclusion

This study provided new data on HL levels among pregnant and preconception women in Vietnam, as well as detailed insights into their awareness of preconception/pregnancy-related nutrition and supplementation. This study also affirms previous observations on the need to consider HL on an individual basis, for example, by not assuming high educational attainment equates to adequate HL. The identification of limited HL and knowledge gaps highlights a need for HCPs and care partners to be aware of the differences in women's HL levels so as to deliver early nutrition and related health messages more effectively.

These insights into women's understanding of key nutrition concepts and beliefs about conception, pregnancy, and childbirth could inform the design of HL-sensitive nutritional education programs and resources to enhance existing parent-focused interventions. With strong partnerships between parents and HCPs to prepare for parenthood, and a wider ecosystem that supports parents' ability to care for their children, the journey to health can begin before conception and continue beyond the “first 1000 days” of life.

## Supplementary Material

Supplemental data

Supplemental data

Supplemental data

Supplemental data

Supplemental data

## Abbreviations Used

HCPs: health care professionals
HL: health literacy
NVS: Newest Vital Sign
