# Supplementary material for: Awareness of Nutrition and Supplements Among Pregnant and Preconception Women: A Real-World Study in Vietnam
Source: Womens Health Rep (New Rochelle). 2023 Oct 25;4(1):506–16. doi: 10.1089/whr.2023.0014 (PMC10615086; doi:10.1089/whr.2023.0014)
Supplement: Supplemental data [file Suppl_Data.pdf]

Supplementary File 1. Consumer research panel methods.

Participants in this study were recruited from a multinational online consumer research panel managed by an established research organisation. Individuals may register to participate in such panels, which are selected to be representative of the included country or countries; if selected, they provide their demographic and certain other profile information that can be used to determine whether they meet the target criteria for a given panel-based study. The registered panellists' profiles are securely stored and kept confidential by the organisation that manages the consumer panel platform. No personally identifiable information is revealed to researchers or research agencies that use the consumer panel, since only anonymous unique panellist IDs are provided for data collection. This system allows for controlled and completely anonymous access for researchers wishing to conduct research with representative panel samples. Upon completing a survey, respondents receive a small non-monetary reward in the form of "panel points", which they can then accumulate and redeem. It is difficult to put a monetary value to these panel points but it is usually in the range of US$3–5 per survey.

For the present sample, the target population was specified as females aged 18–45 residing in 4 major urban centres in Vietnam (Ho Chi Minh City, Hanoi, Da Nang, Can Tho), who were planning to conceive or already pregnant. The recruitment strategy involved a pre-screening step and a quota system that was programmed to achieve the required sample size for each subgroup (exactly 200 pregnant women and 100 pre-conception women). Once the quota for each subgroup was reached by collecting the required number of completed survey responses, additional individuals attempting to complete the online survey were screened out and thanked for their interest in participating. The online questionnaire form was programmed in a way that required respondents to answer all mandatory fields; thus, only complete questionnaires were collected.
