# Supplementary material for: Awareness of Nutrition and Supplements Among Pregnant and Preconception Women: A Real-World Study in Vietnam
Source: Womens Health Rep (New Rochelle). 2023 Oct 25;4(1):506–16. doi: 10.1089/whr.2023.0014 (PMC10615086; doi:10.1089/whr.2023.0014)
Supplement: Supplemental data [file Suppl_TableS1.pdf]

Supplementary Table 1. Respondent demographics by health literacy level

Characteristic	Limited health literacy (N=186)	Possibly limited health literacy (N=98)	Adequate health literacy (N=16)
Age, years			
18–24	9.1%	8.2%	6.3%
25–29	49.5%†‡	32.7%	25.0%
30–34	28.0%	44.9%*	56.3%*
35–39	7.5%	9.2%	12.5%
40–45	5.9%	5.1%	0.0%
City			
Ho Chi Minh City	43.0%	52.0%	75.0%*
Hanoi	45.7%‡	37.8%	18.8%
Da Nang	7.0%	6.1%	6.3%
Can Tho	4.3%	4.1%	0.0%
Household income			
Low	4.8%	2.0%	6.3%
Medium	36.6%	30.6%	25.0%
High	58.6%	67.3%	68.8%
Employment			
Full-time	87.1%	87.8%	87.5%
Part-time	1.6%	5.1%	12.5%
Self-employed	7.0%	6.1%	0.0%
Housewife	3.2%	0.0%	0.0%
Unemployed	1.1%	1.0%	0.0%
Highest educational qualification			
High school	5.9%	3.1%	0.0%
College	10.8%	9.2%	6.3%
Undergraduate	75.3%	81.6%	93.8%*
Postgraduate	8.1%	6.1%	0.0%
Nutritional qualification			
Yes	28.0%‡	19.4%	6.3%
No	72.0%	80.6%	93.8%*

* Significantly different when compared with limited health literacy group
† Significantly different when compared with possibly limited health literacy group
‡ Significantly different when compared with adequate health literacy group
