# Supplementary material for: Awareness of Nutrition and Supplements Among Pregnant and Preconception Women: A Real-World Study in Vietnam
Source: Womens Health Rep (New Rochelle). 2023 Oct 25;4(1):506–16. doi: 10.1089/whr.2023.0014 (PMC10615086; doi:10.1089/whr.2023.0014)

Supplementary Figure 1.
Conception/pregnancy-related nutrition knowledge. (A) Perceived importance of healthy eating habits. (B) Perceived knowledge of healthy eating habits by health literacy level.

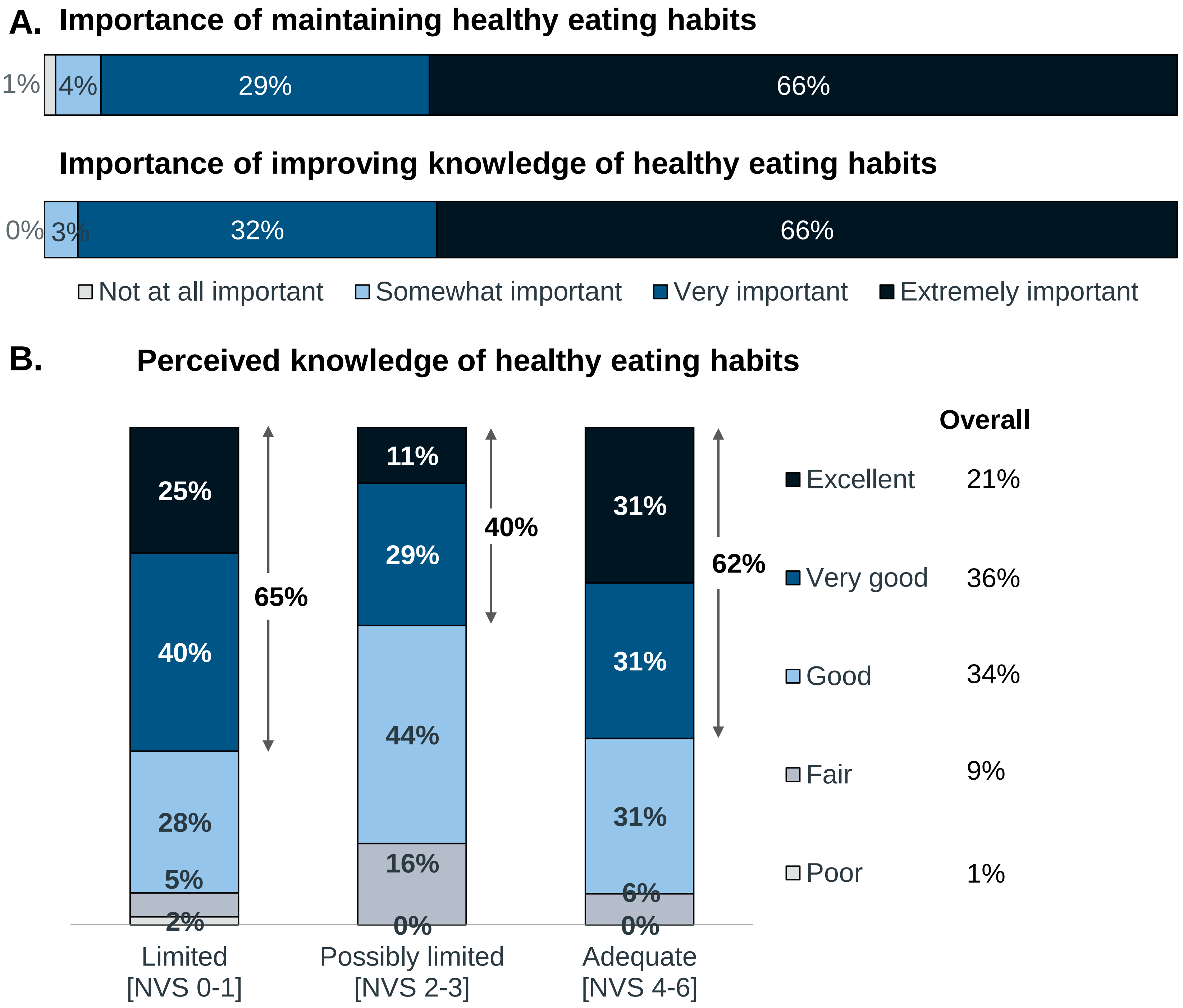

Supplement: Supplemental data [file Suppl_FigureS1.pdf]
