# Supplementary material for: Awareness of Nutrition and Supplements Among Pregnant and Preconception Women: A Real-World Study in Vietnam
Source: Womens Health Rep (New Rochelle). 2023 Oct 25;4(1):506–16. doi: 10.1089/whr.2023.0014 (PMC10615086; doi:10.1089/whr.2023.0014)
Supplement: Supplemental data [file Suppl_FigureS2.pdf]

Supplementary Figure 2. Nutritional supplement usage by pregnancy stage

Current supplement usage according to pregnancy/preconception stage

	Pregnant			Planner	
	Trimester 1	Trimester 2	Trimester 3	Planning – next 12 months	Currently trying to conceive
	N=70	N=93	N=36	N=87	N=13*
**Pre-conception/pregnancy multivitamin ± Folic acid/folate**	42%	57%	75%* †	60%	54%
Iron	54%	66%	72%*	53%	77%
Calcium	41%	57% *	75%* †	45%	46%
Pre-conception/pregnancy multivitamin	30%	51% *	64%*	43%	23%
Multivitamin	28%	37%	36%	40%	23%
DHA / Omega-3/ Fish oil	37%	27%	47%	37%	46%
Vitamin D	24%	29%	31%	26%	31%
Folic acid/ folate	24%	18%	47% * †	33%	31%
Iodine	13%	22%	22%	14%	15%

* Significant when compared with trimester 1 group

† Significant when compared with trimester 2 group
