# Supplementary figures and images for: Awareness of Nutrition and Supplements Among Pregnant and Preconception Women: A Real-World Study in Vietnam
Source: Womens Health Rep (New Rochelle). 2023 Oct 25;4(1):506–16. doi: 10.1089/whr.2023.0014 (PMC10615086; doi:10.1089/whr.2023.0014)

**Supplementary Figure 3.** Sources of nutritional advice or information

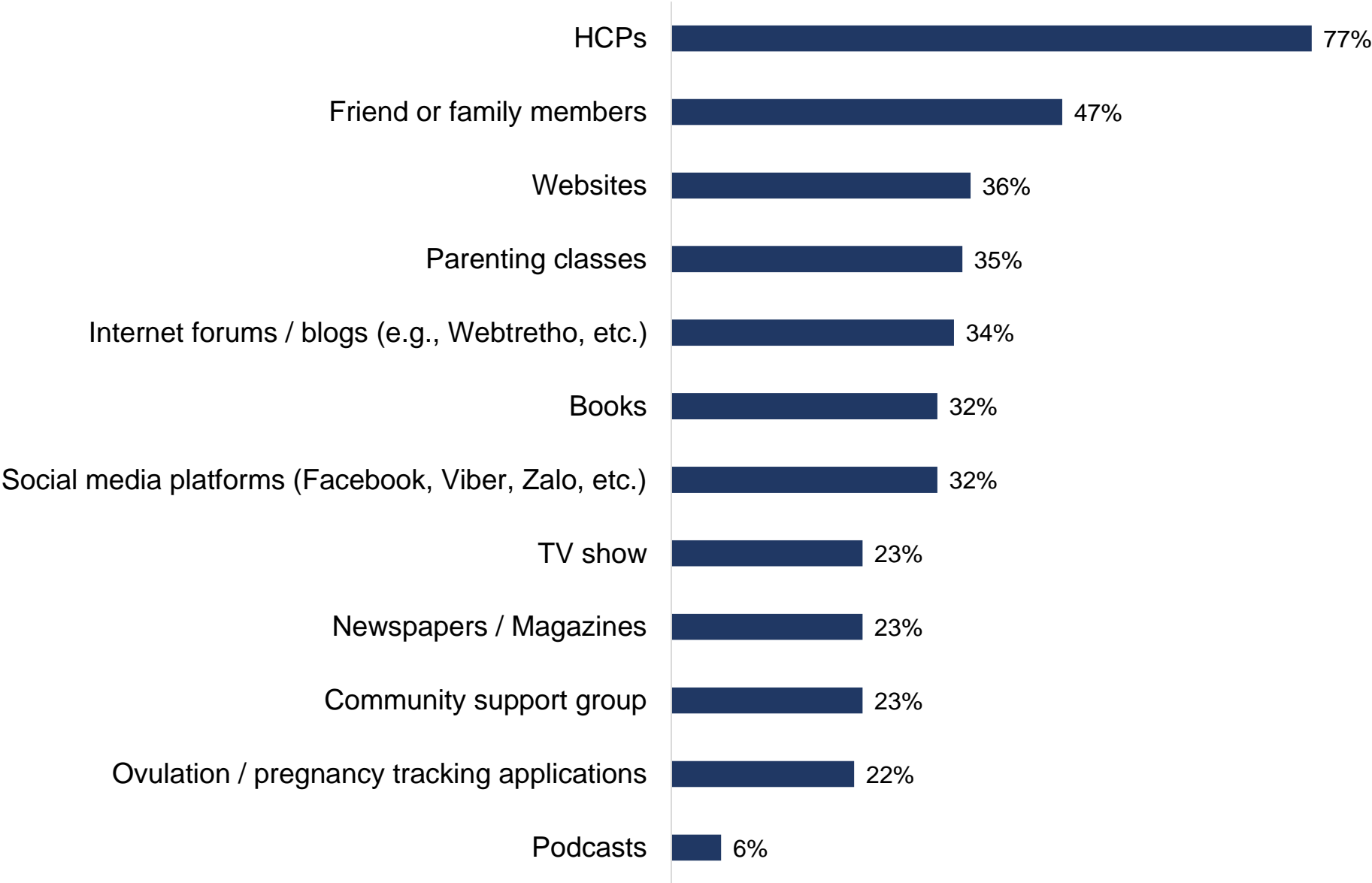

Supplement: Supplemental data [file Suppl_FigureS3.pdf]
